# Lightweight metal surface defect detection algorithm based on pruning and knowledge distillation

**DOI:** 10.1038/s41598-026-57496-0

**Published:** 2026-06-15

**Authors:** Yiqing Cao, Yonger Yao, Lijun Lu

**Affiliations:** 1 College of Intelligent Manufacturing, Putian University, Putian, 351100 Fujian China; 2 School of Mechatronic Engineering and Automation, Shanghai University, Shanghai, 200072 China

**Keywords:** Model pruning, Knowledge distillation, Metal surface defect detection, Industrial inspection, YOLOv8, Engineering, Mathematics and computing

## Abstract

Metal surface defect detection models have large parameters and high computational complexity, making them difficult to deploy on industrial edge devices. To address these issues, this paper proposes a lightweight algorithm with collaborative pruning and distillation. This algorithm, based on YOLOv8, uses L1 regularization structured pruning to compress the SAF-YOLOv8 model for efficiency. The resulting pruned and unpruned models serve as the student and teacher in subsequent knowledge distillation. The BFCD-KD knowledge distillation method fuses middle-layer features with the regression and classification branches of the detection head and the original model loss. This integrated approach transfers teacher knowledge in feature representation, localization, and classification to improve optimization. Experimental results show that this algorithm reduces the model’s FLOPs by 51.0% and parameters by 41.5%, while achieving a mAP@0.50 of 70.6% on the GC10-DET dataset, balancing accuracy and efficiency. In addition, a real-time interactive metal surface defect detection system is developed for industrial sites using the lightweight SAF-YOLOv8 model. The system integrates industrial cameras, a controllable light source, and a graphical interactive interface built on the PyQt5 framework. It supports real-time visualization and interactive defect detection operations.

## Introduction

Against the background of the intelligent upgrading of modern industrial manufacturing, metal surface defect detection is a key step to ensure product quality. Automated schemes based on object detection technology are widely applied in this field. Represented by the YOLO series, one-stage detection models serve as the mainstream methods in this scenario with their end-to-end high efficiency. Detection systems in industrial sites are often deployed on edge devices or mobile terminals with limited computing resources. High-precision models have many parameters and complex structures. They suffer from high computational overhead, large storage requirements, and slow inference speed. As a result, they fail to meet actual deployment requirements^1^. Therefore, on the premise of maintaining high detection accuracy, how to effectively realize model lightweight, reduce computational and storage costs, and improve inference efficiency has become a key issue in the practical deployment of metal surface defect detection technology.

At present, lightweight deep learning methods fall into two categories. The first is designing lightweight network architectures. This approach optimizes convolution operations, network structures, and calculation methods. The goal is to reduce parameter count and computational complexity. Typical models include MobileNet^2–4^, ShuffleNet^5,6^, and EfficientNet^7^. Qian X. et al. propose the LFF-YOLO model with ShuffleNetv2 as the backbone. It combines a lightweight feature pyramid and adaptive receptive field modules. The model also uses focal loss to reduce sample imbalance. Its mAP reaches 79.23% on NEU-DET and 59.78% on GC10-DET. This achieves optimal detection speed while maintaining accuracy^8^. Liang L. et al. propose the SDB-YOLOv8s algorithm with a BS-ShuffleNetV2 backbone. This choice reduces model complexity. They introduce the S-C2f module to suppress redundant information and add a dilated Transformer to better extract contextual and small target features. The mAP increases by 6.4 points on the NEU-DET dataset and by 7.0 points on the Severstal dataset. Parameters and computational complexity drop to 64.8% and 56.2% of the baseline model^9^. Wang B. et al. propose the YOLOMEE algorithm by replacing the YOLOv5 backbone with MobileNetV3, successfully reducing model parameters. They improve feature extraction using the ECA attention mechanism in the Neck layer and speed up bounding box convergence with the EIoU loss function. As a result, model weight, computational complexity, and parameter count fall to 23.3%, 15.8%, and 19.8% of the original values, respectively, while mAP@0.50 reaches 93.8%, demonstrating excellent lightweight performance^10^. The second category adopts model compression technologies for post-optimizing pre-trained models. Without changing the core architecture, it achieves lightweighting by removing redundant parameters and transferring knowledge. Main methods are pruning, quantization, and knowledge distillation. In terms of pruning, Xing Z. et al. propose a collaborative strategy of pruning and fine-tuning to effectively restore the detection accuracy of the pruned model^11^. Jia Y. et al. propose a weight pruning method based on Taylor expansion to reduce model parameter count and improve inference speed^12^. In another approach, Li C. et al. optimize the distillation loss function to strengthen knowledge transfer of defect features^13^. Sabaghian Melika et al. presented a YOLOv8 compression framework integrating dynamic sparse training, structured channel pruning, and channel-wise knowledge distillation. The framework greatly reduces model parameters and computational cost while maintaining high accuracy, and enables real-time aerial object detection on edge devices with TensorRT optimization^14^.

Existing lightweight technologies have made progress, but still have obvious shortcomings in metal surface defect detection. Lightweight network architectures, when compressed, lose feature representation ability, which directly impacts detection accuracy, especially for tiny defects, complex textures, and in noisy environments. During model compression, pruning disrupts the feature integrity established in pre-trained models. These compression effects make it difficult to balance high compression rates with maintaining detection accuracy. As a result, it becomes difficult to balance high compression rates with the maintenance of detection accuracy. Single knowledge distillation offers limited model compression, making it insufficient for edge deployment requirements. These constraints hinder the efficient application of lightweight models in industrial scenarios. Machine tactile sensing methods use physical contact to capture the geometric topography of surface microdefects, providing a non-visual technical pathway for the detection of small defects on metal surface^15^. This paper targets the lightweight and practical deployment needs of metal surface defect detection. It carries out research with collaborative optimization of pruning and knowledge distillation as the core. The SAF-YOLOv8n model^16^ proposed in previous research improves the detection accuracy of metal surface defects by introducing SI-C2f, FASFFHead, and ADown modules. However, these enhancements increase the computational burden, making it difficult for the model to adapt to resource-constrained edge deployment environments. Building on this foundation, the paper takes this model as its research object and, to enhance efficiency, proposes a lightweight improvement scheme through collaborative pruning and distillation. A Binary Classification, Feature, and CIoU Knowledge Distillation (BFCD-KD) loss strategy is proposed. Furthermore, a metal surface defect detection system is developed to validate the practical applicability of the proposed method.

The core work is as follows: (1) The SAF-YOLOv8n model is pruned using the L1-norm filter method to remove redundant filters and reduce parameters and computational cost. (2) The BFCD-KD knowledge distillation method helps the pruned model recover accuracy by using the complete model as the teacher and the pruned model as the student. (3) Training and validation on the GC10-DET dataset assess the collaborative lightweight scheme. (4) By integrating industrial cameras and light sources, an industrial-level defect detection system is built with the lightweight model, demonstrating its feasibility for practical use. Overall, this work presents a new approach for lightweight metal surface defect detection and supports the deployment of such models in industrial and portable scenarios.

## SAF-YOLOv8 model

**Fig. 1:**
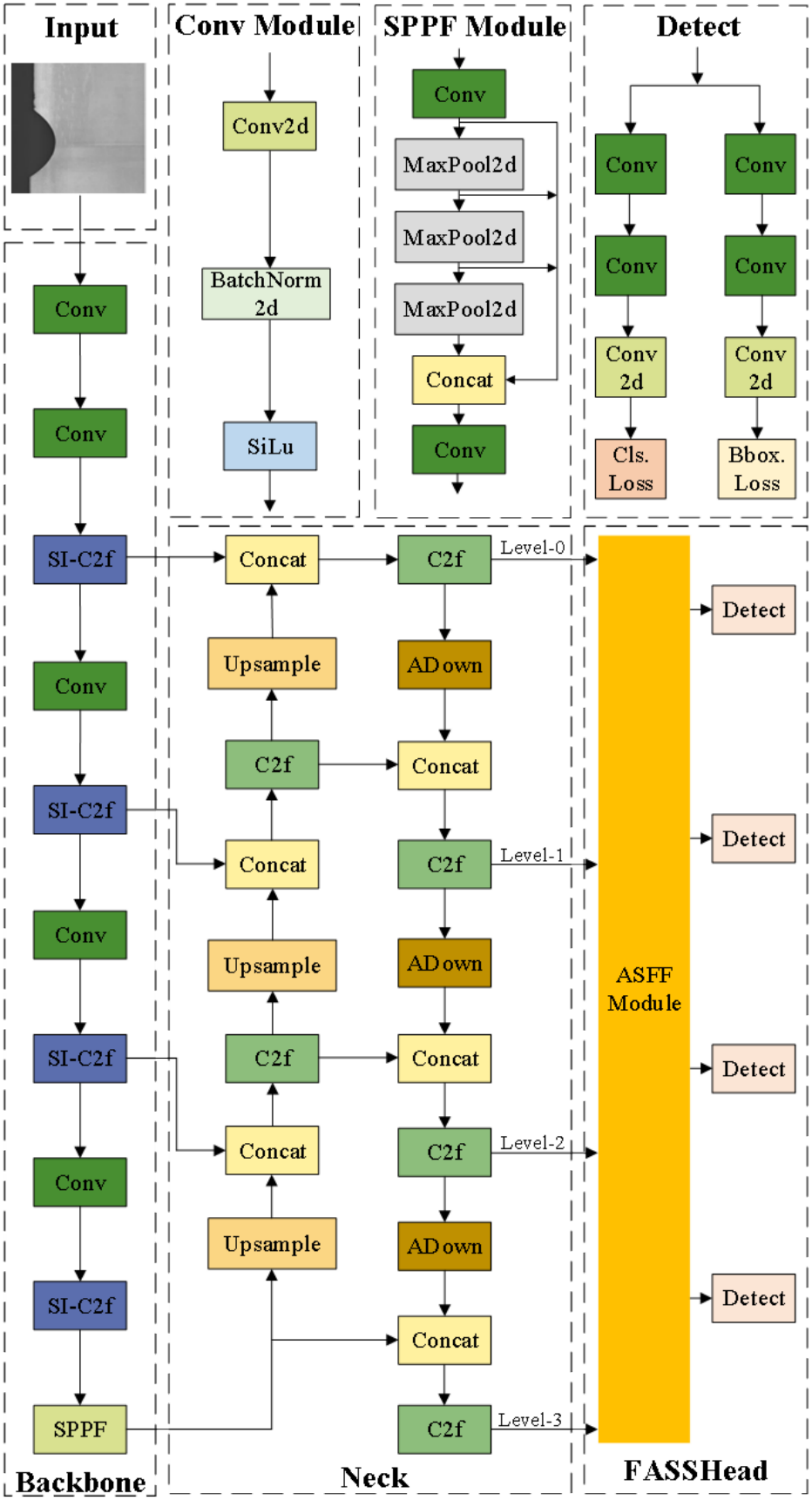
Structure of SAF-YOLOv8 network.

Previous research proposed an improved SAF-YOLOv8 model to address high miss rates and poor multi-scale adaptability in metal surface defect detection. This model was developed based on the GC10-DET dataset^17^. We use YOLOv8n as the baseline model and improve detection accuracy by modifying the backbone network, neck network, and detection head. Figure 1 illustrates the overall structure. Specific improvements are as follows. The improved SI-C2f module is adopted in the backbone network. This module integrates spatial-channel reconstruction convolution. It also includes an iterative attention feature fusion module. As a result, the model’s feature learning and fusion abilities are enhanced. These changes improve the extraction effect for small target defects. The FASFFHead module is introduced in the detection head, and the number of detection heads is extended to 4. Additionally, an adaptive spatial feature fusion mechanism is added to combine information from multiple spatial scales, which strengthens the multi-scale defect detection ability. The ADown adaptive downsampling module is introduced in the neck network. It replaces traditional convolutional downsampling. This reduces the resolution of feature maps but retains key defect features. As a result, information loss during downsampling is reduced.

These improvements enable SAF-YOLOv8 to achieve 70.8% mAP@0.50 on the GC10-DET dataset. This is 3.6 percentage points higher than YOLOv8n and outperforms other YOLO models tested under the same conditions, specifically YOLOv8s, YOLOv10, and YOLOv11, thus improving metal surface defect detection accuracy. This model prioritizes detection accuracy but overlooks deployment constraints on industrial edge devices. Its parameter count and computational cost are 4.1×10⁶ and 14.7×10⁹, both exceeding those of lightweight models commonly used in industrial edge scenarios. In addition, existing model compression technologies have limitations with this model. Single structured pruning can easily remove key feature extraction layers. This causes a sharp drop in detection accuracy. Knowledge distillation also has limited effects on parameter compression and computational cost. Therefore, based on the detection architecture of SAF-YOLOv8, this paper adopts a lightweight design method combining structured pruning and knowledge distillation, effectively reduces the parameters and computational cost on the premise of maintaining the original detection accuracy of the model on the GC10-DET dataset, and finally constructs a lightweight metal surface defect detection model with both high detection accuracy and high edge deployment efficiency.

## Filter pruning based on L1-norm

Pruning technology reduces the weight of convolutional neural networks by removing redundant structured units with low contribution to the network, reduces model parameters and computational complexity, and retains the original model performance as much as possible. To achieve the lightweight goal of the SAF-YOLOv8n model, this paper adopts the L1-norm-based filter pruning method^18^, treats the filter as the basic pruning unit, evaluates the importance of each filter based on its L1-norm weight, and removes redundant filters to achieve model compression. and subsequent fine-tuning is used to alleviate the accuracy loss.

In the convolutional layer, a filter can be represented as a 4-dimensional tensor. Let the number of input channels of the convolutional layer be $C_{in}$, the number of output channels be $C_{out}$, and the filter size be *k × k*. Then the filter corresponding to the *j*-th output channel can be represented as ${\mathbf{w}}_{j} \in {\mathbf{R}}^{{C_{in} \times k \times k}}$, where $j = 1,2, \cdots C_{out}$. The L1-norm of the filter ${\mathbf{w}}_{j}$ is calculated by the formula:

1 $$||{\mathbf{w}}_{j} ||_{1} = \sum\limits_{c = 1}^{{C_{in} }} {\sum\limits_{p = 1}^{k} {\sum\limits_{q = 1}^{k} {|{\mathbf{w}}_{j} \left( {c,p,q} \right)|} } } ,$$

where ${\mathbf{w}}_{j} \left( {c,p,q} \right)$ denotes the weight value of the *j**-th* filter at input channel *c* and spatial position $\left( {p,q} \right)$.

Filter pruning strategies based on the L1-norm are divided into two types according to the range of importance comparisons: local pruning and global pruning. Local pruning sorts and selects filters independently within each convolutional layer, removing filters with the smallest norms according to the layer-wise pruning ratio. This method maintains the structural proportion between layers and is easy to implement, but it may retain filters with low global importance in some layers. Global pruning evaluates filters uniformly across all target layers, determines a unified global threshold $T_{total}$ based on the preset global pruning ratio *α*, and removes all filters whose importance scores are below this threshold. The calculation formula of this threshold is shown as follows:

2 $$\tau = Q_{\alpha } \left( {\left\{ {S_{i,j} |i = 1, \cdots ,I;j = 1, \cdots ,N_{i} } \right\}} \right),$$

where $S_{i,j}$ represents the importance score of the *j**-th* filter in the *i**-th* layer, and *I* represents the total number of convolutional layers involved in global pruning. $N_{i}$ represents the total number of filters in the *i**-th* layer, and $Q_{\alpha } \left( \cdot \right)$ represents the *α*-quantile of the entire set of filter importance scores. After pruning, a fine-tuning stage is performed on the network to recover the representation capability lost due to filter removal, so that the model can maintain the original detection accuracy as much as possible while reducing the number of parameters and computational complexity.

**Fig. 2:**
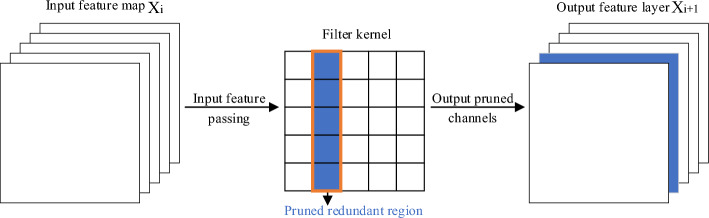
Channel dimension change of layer *i* in filter pruning.

**Fig. 3:**
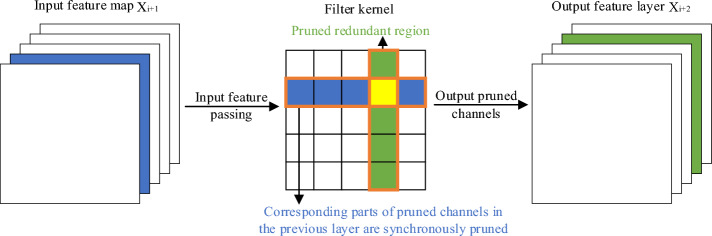
Channel dimension change of layer $i{ + }1$ in filter pruning.

During L1-norm filter pruning, when a filter in a convolutional layer is removed, the corresponding input channels of subsequent convolutional layers must be adjusted synchronously to maintain the structural integrity of network forward propagation^18^. Figure 2 shows the channel dimension change of the *i**-th* layer during filter pruning, where ${\mathbf{X}}_{i}$ is the input feature map of the *i**-th* layer, and the blue part in the figure represents the pruned filters of this layer; the number of output channels of the output feature map ${\mathbf{X}}_{{i{ + }1}}$ of this layer is reduced accordingly. All filters in the $\left( {i + 1} \right)$*-th* layer take the output channels of the *i**-th* layer as input, and each filter must remove all convolution kernel slices corresponding to the pruned output channels. Figure 3 shows the channel dimension change of the $\left( {i + 1} \right)$*-th* layer during filter pruning, where ${\mathbf{X}}_{{i{ + }1}}$ is the output feature of the *i**-th* layer, the blue part in the figure represents the channels corresponding to the filters deleted in the *i**-th* layer, the middle matrix represents the convolution kernel of the next layer, and the blue part is the weights to be deleted corresponding to ${\mathbf{X}}_{{i{ + }1}}$; the adjusted output feature map ${\mathbf{X}}_{i + 2}$ of the $\left( {i + 1} \right)$*-th* layer is obtained on the final right side. This adjustment ensures that the pruned network still maintains correct tensor dimensions and information structure.

## Knowledge distillation-based metal surface defect detection method

**Fig. 4:**
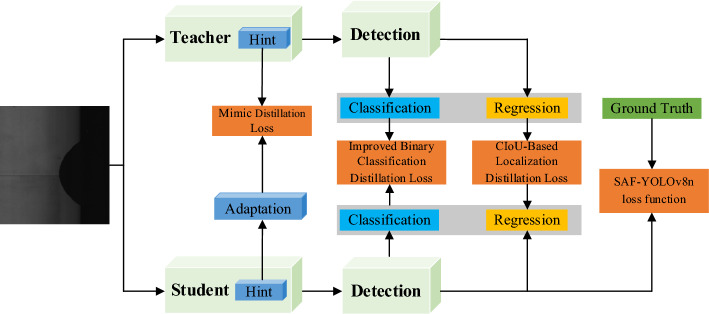
Overall architecture of BFCD-KD.

To address the accuracy drop caused by pruning, a knowledge distillation method named BFCD-KD is proposed, where the pre-pruned SAF-YOLOv8n serves as the teacher and the post-pruned model as the student. This method transfers defect features via feature map alignment, adapts to detection head outputs and enhances hard example learning through difference-aware weighted binary classification distillation, and compensates for localization accuracy loss using CIoU-based object localization distillation. The overall architecture of BFCD-KD is shown in Fig 4.It learns knowledge from the teacher model through three dimensions: the Mimic distillation loss, the improved binary classification distillation loss, and the CIoU-based localization distillation loss. Meanwhile, it combines the original loss function of SAF-YOLOv8n to achieve a balance between accuracy and lightweight performance.

### Feature-based distillation

This paper selects feature layers with the same spatial size and channel dimensions in the teacher and student models to facilitate alignment. The Mimic method^19^ is used to design the feature distillation loss. The mean square errors of the corresponding feature maps of the teacher and student models are calculated to constrain the student model to reproduce the middle-layer feature representation of the teacher model, transfer deep semantic feature extraction, and improve the recognition of small metal surface defects. The feature distillation loss function is defined as follows:

3 $${\mathcal{L}}_{Mimic} = \sum\limits_{i = 1}^{K} {\frac{1}{{N \cdot C_{i} \cdot H_{i} \cdot W_{i} }}||F_{T,i} - F_{S,i} ||_{F}^{2} }$$

where *K* is the number of feature layers involved in alignment; ${\mathbf{F}}_{T,i}$ is the feature map of the *i**-th* layer in the teacher model, and ${\mathbf{F}}_{S,i}$ is the feature map of the *i**-th* layer in the student model, both of which have dimensions of $N \times C_{i} \times H_{i} \times W_{i}$ (where *N* is the batch size, and $C_{i}$,$H_{i}$ and $W_{i}$ are the number of channels, height, and width of the *i**-th* layer feature map, respectively); $|| \cdot ||_{F}^{2}$ is the square of the Frobenius norm of the matrix, representing the element-wise sum of squares of the feature map differences, and the calculation formula is:

4 $$||{\mathbf{F}}_{T,i} - {\mathbf{F}}_{S,i} ||_{F}^{2} { = }\sum\limits_{n = 1}^{N} {\sum\limits_{c = 1}^{{C_{i} }} {\sum\limits_{h = 1}^{{H_{i} }} {\sum\limits_{w = 1}^{{w_{i} }} {\left( {{\mathbf{F}}_{T,i}^{{\left( {n,c,h,w} \right)}} - {\mathbf{F}}_{S,i}^{{\left( {n,c,h,w} \right)}} } \right)} } } }^{2} ,$$

where ${\mathbf{F}}_{T,i}^{{\left( {n,c,h,w} \right)}}$ and ${\mathbf{F}}_{S,i}^{{\left( {n,c,h,w} \right)}}$ respectively denote the scalar element values of the *n-th* sample, *c-th* channel, and spatial position $\left( {h,w} \right)$ in the *i-th* layer feature map of the teacher model and the student model. Combined with Formulas (3) and (4), the loss function calculates the sum of squares of the differences between the corresponding elements of the feature maps and normalizes the result by dividing it by the total number of elements $N \cdot C_{i} \cdot H_{i} \cdot W_{i}$ of the *i*-*th* layer feature map. This process removes the effects of spatial size, channel number, and batch size on the loss value. As a result, the loss magnitude remains stable across feature maps of varying scales, enabling the optimizer to update parameters more effectively during model training. This method efficiently guides the student model to match the teacher model’s middle-layer representation in visual tasks.

### Detection head-based distillation

The detection head of SAF-YOLOv8 is composed of a classification branch and a regression branch. The classification branch predicts the class confidence of target defects to realize accurate discrimination of defect types. The regression branch focuses on the regression calculation of bounding box coordinates to locate the position and size of defects. To better transfer high-quality knowledge in defect detection, we design improved binary classification and CIoU-based localization distillation losses, both built on a two-branch detection head structure. This method enhances the student model by smoothing learning objectives with the teacher model’s soft label predictions for class confidence and bounding box positions. This leads to improved classification accuracy, localization precision, and understanding of data uncertainty, resulting in better overall performance. The total distillation loss function is defined as follows:

5 $${\mathcal{L}}_{total}^{dis} \left( x \right) = \alpha_{1} {\mathcal{L}}_{cls}^{dis} \left( x \right) + \alpha_{2} {\mathcal{L}}_{loc}^{dis} \left( x \right)$$

In the above formula, adjustable weight hyperparameters $\alpha_{1}$ and $\alpha_{2}$ dynamically balance the classification and localization distillation losses in the total loss, enabling adaptation to different defect detection performance requirements. ${\mathcal{L}}_{cls}^{dis} \left( x \right)$ and ${\mathcal{L}}_{loc}^{dis} \left( x \right)$ are the improved binary classification distillation loss and CIoU-based localization distillation loss, respectively.

#### Improved binary classification distillation loss

In the SAF-YOLOv8 detection architecture, the classification head uses the Sigmoid activation function. It takes the raw prediction Logits from the model’s last layer without applying any activation. Using Sigmoid activation, the model breaks multi-class classification into independent binary problems. Traditional knowledge distillation methods rely on Softmax and KL divergence, which assume class mutual exclusiveness, making them incompatible with this approach. Using Sigmoid activation, the model breaks multi-class classification into independent binary problems. Traditional knowledge distillation methods rely on Softmax and KL divergence, which assume class mutual exclusiveness, making them incompatible with this approach. Direct application to Sigmoid outputs ignores class independence. Later in distillation, the student’s predictions on many simple background samples closely match those of the teacher model. Distillation with equal weights will make the small gradients generated by simple samples drown out the effective gradients of hard samples, reducing the efficiency of knowledge transfer. This paper proposes an improved Difference-Aware Weighted Binary Classification Distillation Loss (DW-BCDL), based on Binary Classification Distillation Loss (BCDL)^20^, to address the above problems. The original BCDL can adapt to distribution alignment under Sigmoid activation, but it does not consider the imbalance between easy and hard samples. Therefore, this paper introduces an adaptive weighting mechanism to improve the stability of the distillation process.

Let ${\mathbf{Z}}^{t} \in {\mathbf{R}}^{G \times N}$ and ${\mathbf{Z}}^{{\mathrm{s}}} \in {\mathbf{R}}^{G \times N}$ denote the output logits of the teacher and student models, respectively, with *G* as the number of anchor points and *N* as the number of classes. The Sigmoid function maps Logits to the interval $\left( {0,1} \right)$, yielding the binary classification probabilities of the student model and the teacher model as $p_{i,j}^{s} = \sigma \left( {{\mathbf{Z}}_{i,j}^{s} } \right)$ and $p_{i,j}^{t} = \sigma \left( {{\mathbf{Z}}_{i,j}^{t} } \right)$, where $i \in \left[ {1,G} \right],j \in \left[ {1,N} \right]$. To address the issue that traditional binary cross-entropy loss does not account for the imbalance between easy and hard samples, this paper proposes the DW-BCDL method. First, an adaptive weight term $w_{i,j}$ is designed. This weight term is defined as an exponential function of the absolute difference between the teacher’s and student’s prediction probabilities. When the student model’s prediction is very close to the teacher model’s ($|p_{i,j}^{t} - p_{i,j}^{s} | \approx 0$), the sample is considered a simple sample, and its distillation weight is automatically reduced. Conversely, when there is a significant divergence between the two, the weight increases, forcing the student model to approach the teacher’s soft labels through larger gradient updates. This mechanism is expressed as $w_{i,j} { = }|p_{i,j}^{t} - p_{i,j}^{s} |^{\beta }$, where *β* is a focusing hyperparameter that adjusts attention to divergent samples. Combining the above weight term with binary cross-entropy, the classification distillation loss function is given by:

6 $${\mathcal{L}}_{cls}^{dis} \left( x \right) = \sum\limits_{i = 1}^{G} {\sum\limits_{i = 1}^{N} {w_{i,j} \cdot {\mathcal{L}}_{BCE} \left( {p_{i,j}^{s} ,p_{i,j}^{t} } \right)} }$$

This formula uses the teacher’s soft label $p_{i,j}^{t}$ to transfer knowledge and dynamically adjusts the learning intensity of each class channel via a difference-aware weighting term. In the above formula, the calculation formula of ${\mathcal{L}}_{BCE} \left( {p_{i,j}^{s} ,p_{i,j}^{t} } \right)$ is:

7 $${\mathcal{L}}_{BCE} \left( {p_{i,j}^{s} ,p_{i,j}^{t} } \right) = - p_{i,j}^{t} \log \left( {p_{i,j}^{s} } \right) - (1 - p_{i,j}^{t} )\log \left( {1 - p_{i,j}^{s} } \right)$$

In this way, DW-BCDL can achieve distribution alignment under the Sigmoid architecture and effectively alleviate the imbalance between positive and negative samples, as well as between easy and hard samples, through an adaptive weighting mechanism, thereby improving the accuracy of classification knowledge distillation.

#### CIoU-based localization distillation loss

To more comprehensively quantify the spatial deviation between the teacher and student models’ bounding boxes, this paper proposes a CIoU-based localization distillation loss applied to the continuous coordinate layer after DFL decoding in the regression branch of SAF-YOLOv8. The loss function is defined as follows:

8 $${\mathcal{L}}_{loc}^{dis} \left( x \right) = \sum\limits_{i = 1}^{G} {\max \left( {w_{i,j} } \right) \cdot \left( {1 - CIoU\left( {b_{i}^{t} ,b_{i}^{s} } \right)} \right)}$$

where *G* is the total number of anchor points generated by SAF-YOLOv8 for a single input image. ${\mathbf{b}}_{i}^{t}$ and ${\mathbf{b}}_{i}^{s}$ are the continuous bounding box coordinates of the teacher model and the student model at the *i*-*th* anchor point after DFL decoding, respectively. ${\mathrm{CIoU}}\left( {{\mathbf{b}}_{i}^{t} ,{\mathbf{b}}_{i}^{s} } \right)$ is the complete intersection over union of the two bounding boxes, and $1 - {\mathrm{CIoU}}\left( {{\mathbf{b}}_{i}^{t} ,{\mathbf{b}}_{i}^{s} } \right)$ is used to quantify the spatial deviation of the teacher and student bounding boxes in position, size, and shape. $\max \left( {w_{i,j} } \right)$ is the sample importance weight of the *i*-*th* anchor point, which is obtained by taking the maximum value of the weight values of this anchor point across all class dimensions. The goal of this loss is to minimize ${\mathcal{L}}_{loc}^{dis} \left( x \right)$, so that the student model’s predicted bounding boxes are consistent with those of the teacher model in spatial position, size, and shape, thereby enabling localization knowledge transfer.

## Experimental results and analysis of model pruning and knowledge distillation for SAF-YOLOv8n

The experimental evaluation metrics include Precision (P), Recall (R), Average Precision (AP), mean Average Precision (mAP), number of parameters and computational complexity (FLOPs). The calculation formulas of Precision, Recall, Average Precision, and mean Average Precision are given as follows:

9 $$P = \frac{TP}{{TP + FP}}$$

10 $$R = \frac{TP}{{TP + FN}}$$

11 $$AP = \int_{0}^{1} P \left( R \right)dR$$

12 $$mAP = \frac{1}{N}\sum\limits_{i = 1}^{N} {AP_{i} } .$$

In Equations (9)-(12), *TP* and *FP* denote the number of true positive samples and false positive samples, respectively, while FN denotes the number of false negative samples. *AP* is defined as the area under the Precision-Recall curve. *N* represents the total number of classes, and *AP*_*i*_ denotes the average precision of the *i*-*th* class, where *i* is the class index. mAP@0.50 refers to the mean AP at an IoU threshold of 0.50. mAP@0.50:0.95 refers to the mean AP calculated over IoU thresholds ranging from 0.50 to 0.95 with a step size of 0.05.

### Experimental results and analysis of model pruning

The basic parameters of pruning training are as follows: cross-layer global pruning is enabled, the SGD optimizer is adopted, the input image size is 640×640, the batch size is 16, and the total training epoch is 300. Mosaic data augmentation is applied throughout training to improve the model’s robustness. The experimental environment configuration is shown in Table 1.

**Table 1:** Experimental environment configuration.

Experimental environment	Configuration
Operating system	Ubuntu 22.04
CPU	Intel Xeon Platinum 8352V
GPU	NVIDIA GeForce RTX 4090
CUDA Version	CUDA 12.1
Deep learning framework	PyTorch 2.1.0
Compilation language	Python 3.10

**Fig. 5:**
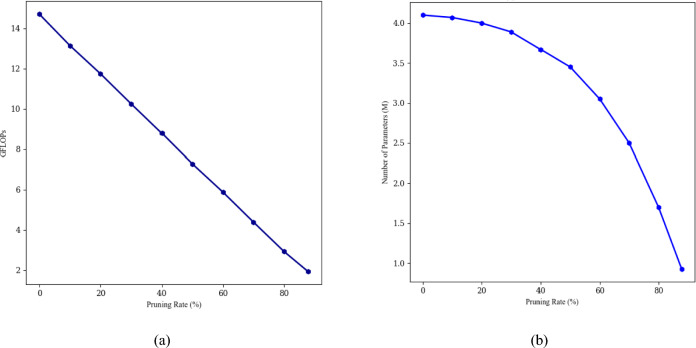
Trend of computational complexity and parameter quantity with pruning rate. (**a**) trend of computational complexity with pruning rate, (**b**) trend of parameter quantity with pruning rate.

The pruning rate is defined as the ratio of the removed FLOPs to the total FLOPs of the original model. Figure 5 shows the trends in computational complexity and the number of parameters with increasing pruning rate. As the pruning rate increases, the model’s computational complexity decreases approximately linearly, while the number of parameters declines nonlinearly, resulting in a significant reduction in model size.

To balance pruning efficiency and detection accuracy, two pruning rates, 33.3% and 50.0%, are selected for evaluation. As shown in Fig. 5, the computational cost decreases approximately linearly with increasing pruning rate, while the parameter reduction exhibits a clear inflection around 50.0%. The 33.3% setting provides a moderate trade-off between efficiency and performance, whereas 50.0% serves as an upper bound that maximizes compression benefits without severely compromising feature representation. Excessive pruning may still degrade performance to a level unsuitable for industrial inspection tasks, even with knowledge distillation.

Table 2 lists the data after pruning of the SAF-YOLOv8 model. Before pruning, the FLOPs and the number of parameters of the SAF-YOLOv8n model are 4.1×10⁶ and 14.7×10⁹, respectively. At a pruning rate of 33.3%, the FLOPs and the number of parameters of the SAF-YOLOv8n model are compressed to 3.8×10⁶ and 9.7×10⁹, respectively, with mAP@0.50 reaching 68.7%, which is higher than that of the YOLOv8n model, indicating that moderate pruning can balance efficiency and accuracy. At a pruning rate of 50%, the FLOPs, the number of parameters, and the model size are 2.4×10⁶, 7.2×10⁹, and 5.3 MB, respectively, which are reduced by 20%, 11% and 11% compared with the YOLOv8 model, and the mAP@0.50 is 67.5%, outperforming the latter. The above experimental results demonstrate that the pruning method proposed in this paper can significantly reduce model redundancy and computational load while effectively maintaining model detection accuracy, laying a foundation for subsequent distillation compensation.

**Table 2:** Pruning results of SAF-YOLOv8n.

Model	mAP0.50 (%)	mAP@0.50-0.95 (%)	Precision (%)	Recall (%)	Parameters (10^6^)	FLOPs (10^9^)	Model Size (MB)
YOLOv8n	67.2	33.0	67.7	67.1	3.0	8.1	5.94
SAF-YOLOv8n	70.8	35.4	76.4	62.0	4.1	14.7	8.7
SAF-YOLOv8n (Pruning rate: 33.3%)	68.7	32.5	71.0	67.8	3.8	9.7	8.2
SAF-YOLOv8n (Pruning rate: 50.0%)	67.5	32.4	63.9	68.3	2.4	7.2	5.3

**Fig. 6:**
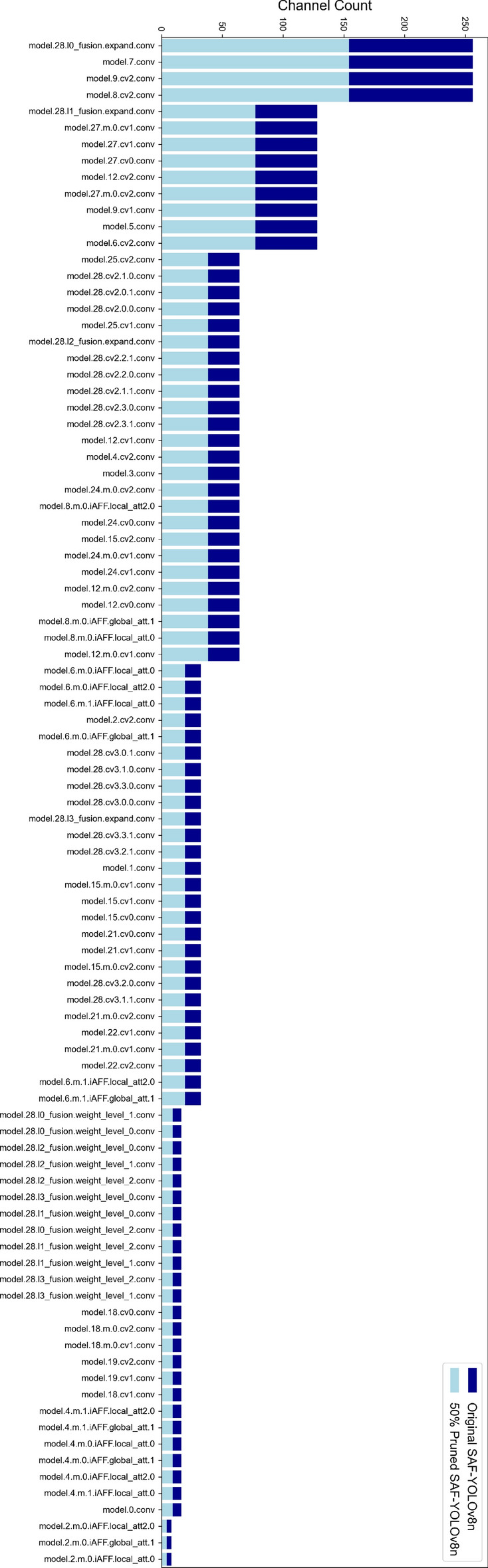
Channel comparison diagram of SAF-YOLOv8n before and after pruning.

Figure 6 shows the channel comparison before and after pruning of the SAF-YOLOv8n model. Dark blue bars represent channel numbers in the original SAF-YOLOv8n, and light blue bars represent those in the model with a 50% pruning rate. Only layers with changed channel numbers are shown. To avoid destroying the core feature extraction and prediction logic, key functional layers are skipped based on the module characteristics of SAF-YOLOv8n. The terminal convolutional layer of the detection head, the DFL layer, and the FASFF multi-scale fusion weight convolutional layer are strongly coupled with the detection task. Their channel dimensions correspond to defect class confidence, bounding box offsets, and multi-scale fusion weights. They are output core dimension constraint layers. Pruning would break the output dimension matching and cause detection failure. In the backbone network, the group normalization layer in the spatial reconstruction unit (SRU) and the channel compression convolutional layer in the channel reconstruction unit (CRU) are core units for feature reconstruction and enhancement. Pruning would interrupt the feature reconstruction process and weaken the feature extraction ability for tiny defects and weak-texture defects. Therefore, these layers are retained during channel pruning, and only redundant feature convolutional layers are pruned. This achieves model lightweighting while preserving core defect representation and prediction ability, ensuring stable detection performance.

### Experimental results and analysis of knowledge distillation

For knowledge distillation, the classification distillation loss weight $\alpha_{1}$ and localization distillation loss weight $\alpha_{2}$ are both set to 1.0, and the focusing parameter *β* of the DW-BCDL loss function is set to 1.0. The experimental environment remains the same as model pruning.

The SAF-YOLOv8n model is pruned by 50%, and the results of the distillation experiments are shown in Table 3. When only feature-based Mimic distillation is used, the model’s mAP@0.50 and mAP@0.50-0.95 are 70.1% and 33.7%, respectively. These values are 2.6 percentage points and 1.3 percentage points higher than those of the pruned model. When only detection-head-based distillation is used, the model’s mAP@0.50 is 68.4%, which is 0.9 percentage points higher than the pruned model’s. Both distillation methods help to restore the accuracy of the pruned model. The BFCD-KD distillation method combines Mimic distillation, detection-head distillation, and the SAF-YOLOv8 loss function. The number of parameters and FLOPs of the distilled model are reduced by 41.5% and 51.0% respectively, compared with SAF-YOLOv8n. The mAP@0.50 and mAP@0.50-0.95 are 70.6% and 34.8% respectively, which are only 0.2 and 0.6 percentage points lower than those of SAF-YOLOv8n. In addition, the number of parameters and FLOPs of the distilled model are reduced by 20.0% and 11.1% respectively, compared with YOLOv8n. The mAP@0.50 and mAP@0.50-0.95 are increased by 3.4 and 1.8 percentage points, respectively. Experimental results show that the BFCD-KD distillation method effectively compensates for the accuracy loss caused by pruning without increasing model complexity and achieves collaborative optimization between lightweight and accuracy.

**Table 3:** Distillation experiment results of the pruned model.

Model	mAP0.50 (%)	mAP@0.50-0.95 (%)	Precision (%)	Recall (%)	Parameters(10^6^)	FLOPs(10^9^)
YOLOv8n	67.2	33.0	67.7	67.1	3.0	8.1
SAF-YOLOv8n	70.8	35.4	76.4	62.0	4.1	14.7
SAF-YOLOv8n (Pruning rate: 50.0%)	67.5	32.4	63.9	68.3	2.4	7.2
Feature Distillation	70.1	33.7	74.1	60.3	2.4	7.2
Detection Head Distillation	68.4	32.9	63.4	67.8	2.4	7.2
BFCD-KD	70.6	34.8	69.6	68.6	2.4	7.2

**Fig. 7:**
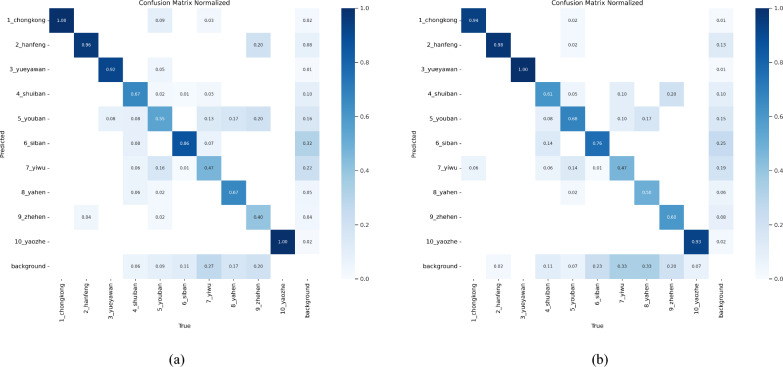
Comparison of confusion matrices: (**a**) model after knowledge distillation, (**b**) SAF-YOLOv8n with 50% pruning rate.

Figure 7 shows the comparison of the confusion matrices between the pruned SAF-YOLOv8n model and the model after knowledge distillation. Figure 7(a) shows the confusion matrix of the distilled model. Figure 7(b) shows the confusion matrix of the model with a 50% pruning rate. The comparison shows that the correct classification rates for punching, water spot, silk spot, rolled pit, and waist folding defects increase by 0.06, 0.06, 0.10, 0.17, and 0.07, respectively, after distillation. The correct classification rates for punching and waist folding defects are 1.00. This indicates that the distillation method effectively alleviates the accuracy loss caused by pruning.

### Analysis of comparative experimental results

The superiority of the BFCD-KD distilled model is verified by comparison with various YOLO models. The results are shown in Table 4. The distilled model clearly outperforms YOLOv5s, YOLOv8n, YOLOv8s, YOLOv10n, YOLOv10s, YOLOv11s, YOLOv12s, and YOLOv13s by having fewer parameters, lower FLOPs, and superior mAP@0.50: improvements are 1.0, 3.4, 1.4, 11.7, 8.0, 2.2, 4.4, and 1.5 percentage points, respectively. It clearly outperforms YOLOv11n, YOLOv12n, and YOLOv13n, with the distilled model’s FLOPs exceeding theirs by 0.9×10⁹, 0.9×10⁹, and 1.0×10⁹, respectively. But its mAP@0.50 increases by 5.3, 6.6, and 2.1 percentage points, respectively. In summary, the BFCD-KD model remains lightweight and achieves high detection accuracy.

**Table 4:** Comparative experiments with different models.

Model	mAP@0.5 (%)	mAP@0.5:0.95 (%)	Precion (%)	Recall (%)	Parameters (10^6^)	FLOPs (10^9^)
YOLOv5s	69.6	33.0	67.5	70.2	7.0	15.8
YOLOv8n	67.2	33.0	67.7	67.1	3.0	8.1
YOLOv8s	69.2	33.6	70.5	63.3	11.1	28.5
YOLOv10n	58.9	29.9	59.9	56.4	2.7	8.2
YOLOv10s	62.6	30.4	59.9	59.6	8.0	24.5
YOLOv11n	65.3	32.7	69.5	63.7	2.6	6.3
YOLOv11s	68.4	34.2	72.1	64.2	9.4	21.3
YOLOv12n	64.0	31.0	62.3	67.2	2.6	6.3
YOLOv12s	66.2	33.0	65.8	64.4	9.2	21.2
YOLOv13n	68.5	32.8	60.1	70.9	2.4	6.2
YOLOv13s	69.1	34.9	74.4	63.9	9.0	20.7
BFCD-KD	70.6	34.8	69.6	68.6	2.4	7.2

### Simulation experiments based on lightweight model

**Fig. 8:**
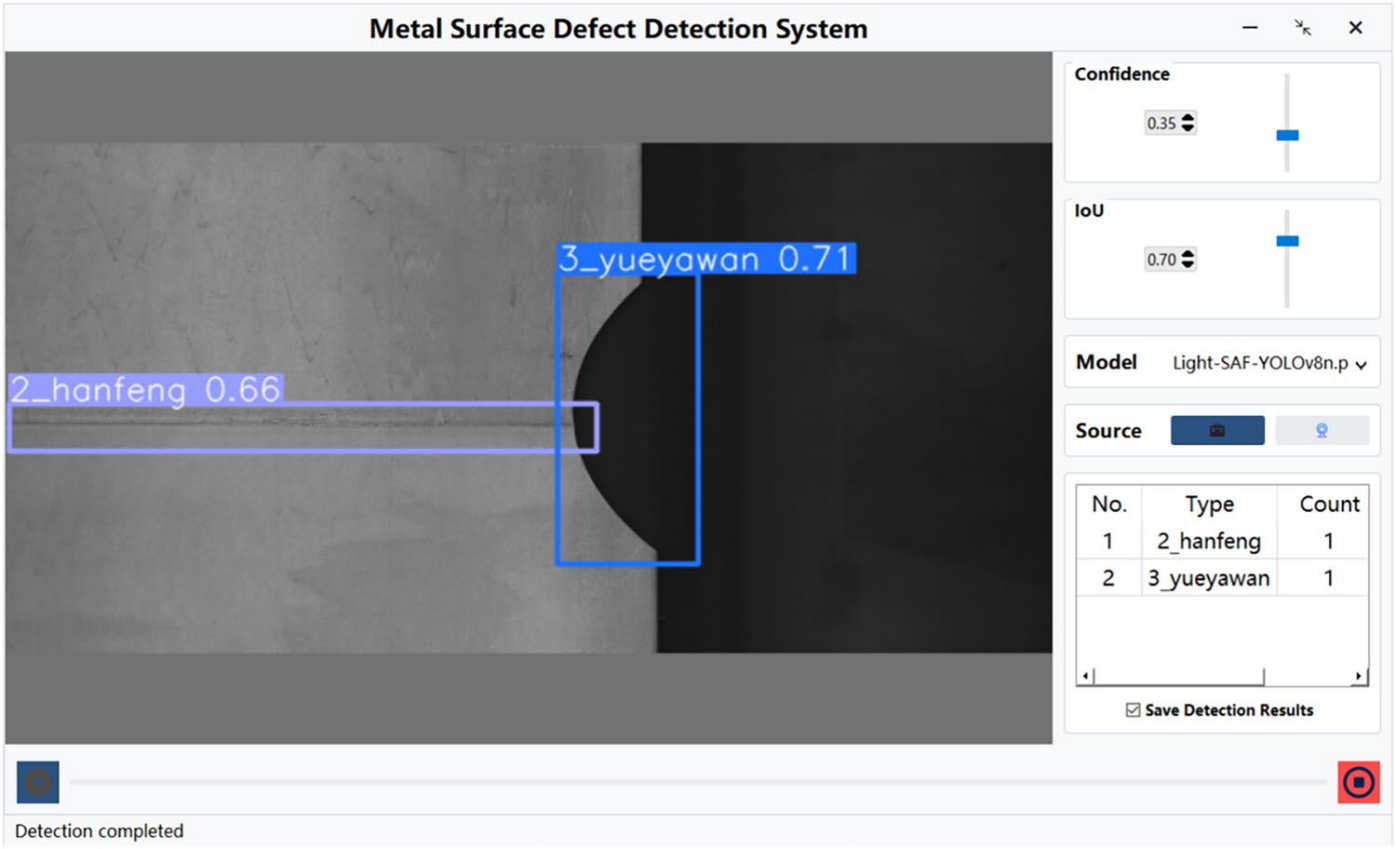
Visual interface for defect recognition of experimental dataset.

**Fig. 9:**
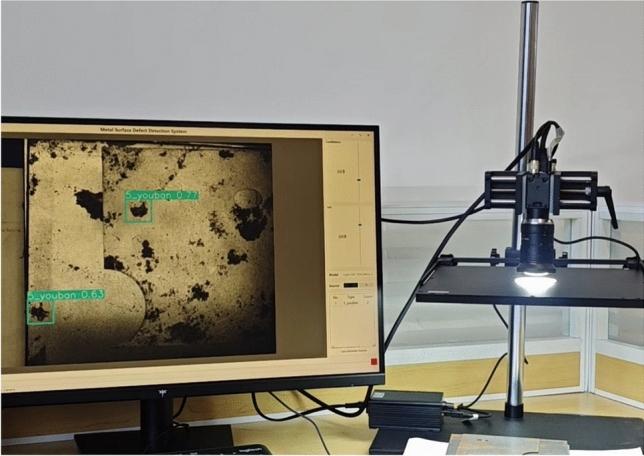
Detection of the metal surface defect system.

**Fig. 10:**
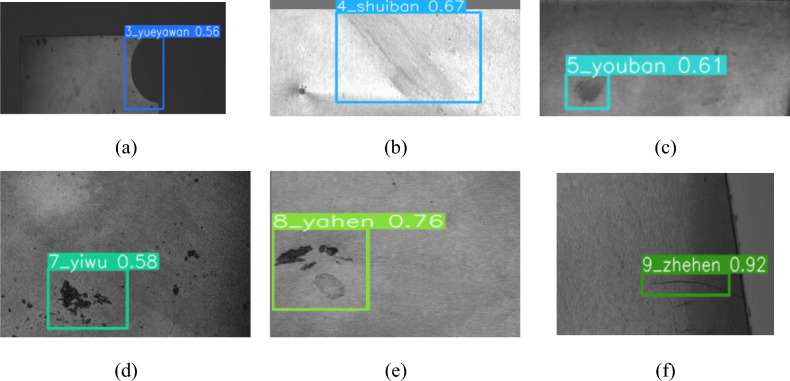
Detection results of self-made dataset: (**a**) crescent gap, (**b**) water spot, (**c**) oil spot, (**d**) inclusion, (**e**) rolled pit and (**g**) crease.

The GC10-DET dataset used in Section "Experimental Results and Analysis of Model Pruning and Knowledge Distillation for SAF-YOLOv8n" is a public benchmark for metal surface defect detection, covering ten typical defect types such as punching, water spot, and silk spot. It is used for model training and performance evaluation. The self-constructed dataset in this section includes similar defect categories, such as crescent gap, rolled pit, and crease, and is built as an extension for engineering validation. It is collected from a self-built experimental hardware platform developed in this study to evaluate the end-to-end performance of the lightweight model under practical deployment conditions, demonstrating its feasibility and applicability in real-world inspection scenarios. A visual interactive interface for detecting metal surface defects is developed using the PyQt5 framework, based on the above lightweight model. The interface supports real-time adjustment of confidence and IoU thresholds. Users can load the Light-SAF-YOLOv8 lightweight model and input either local images or data from industrial cameras. Figure 8 shows the defect recognition results for the experimental dataset on this interface. The number of each defect type is counted on the right side. The saving of detection results is supported. With this visual interactive interface and image acquisition hardware, an integrated hardware-software system for detecting metal surface defects is constructed. Figure 9 shows the physical system and its detection effect. The system consists of an industrial camera, a lighting source, a metal plate to be detected, and visual detection software. It enables full-process automation for defect detection. Figure 10 shows the system’s detection results on the self-made dataset of metal surface defects. The dataset covers six typical defects, including crescent gap, water spot, oil spot, inclusion, rolled pit, and crease. Experimental results show that the system can effectively identify a wide range of defects. The detection confidence of the crease defect is 0.92. The effectiveness and engineering practicability of the detection system are verified.

## Conclusion

This paper focuses on the trade-off between lightweight and performance in metal surface defect detection models. A collaborative optimization strategy of pruning and distillation is proposed. The SAF-YOLOv8n model is compressed, and its performance is compensated. L1 regularization pruning reduces the model’s parameter count and FLOPs by 41.46% and 51.02%, respectively. The BFCD-KD fusion distillation strategy realizes multi-dimensional knowledge collaborative transfer. The model’s mAP@0.50 is 70.6%. It is only 0.2 percentage points lower than that of the original model. The balance between accuracy and efficiency is achieved. Compared with directly training a lightweight model, our strategy of first expanding training and then compressing optimization enables the lightweight model to inherit higher-quality high-level semantic features, effectively mitigating the accuracy loss caused by model compression. Experimental results show that the collaborative strategy of pruning and fusion distillation reduces the performance loss of lightweight models. It provides a promising technical solution for high-precision defect detection on industrial edge devices..

In future work, we plan to address the background noise introduced by global feature distillation by incorporating an attention mask mechanism to guide the model to focus on defect regions, thereby improving distillation effectiveness. Subsequent research will explore the integration of model quantization and multi-teacher knowledge distillation for further compression, as well as the application of lightweight Transformer architectures for defect detection. Moreover, we will investigate the deployment and optimization of the model on edge devices in industrial production lines to enhance its practical applicability in real-world scenarios.

## Data Availability

The datasets used and/or analyzed during the current study available from the corresponding author on reasonable request.
